# Genetic dissection of morphological variation between cauliflower and a rapid cycling *Brassica oleracea* line

**DOI:** 10.1093/g3journal/jkad163

**Published:** 2023-08-28

**Authors:** Lan Shuan Shuang, Hugo Cuevas, Cornelia Lemke, Changsoo Kim, Tariq Shehzad, Andrew H Paterson

**Affiliations:** Plant Genome Mapping Laboratory, University of Georgia, Athens, GA 30602, USA; Plant Genome Mapping Laboratory, University of Georgia, Athens, GA 30602, USA; Plant Genome Mapping Laboratory, University of Georgia, Athens, GA 30602, USA; Plant Genome Mapping Laboratory, University of Georgia, Athens, GA 30602, USA; Plant Genome Mapping Laboratory, University of Georgia, Athens, GA 30602, USA; Plant Genome Mapping Laboratory, University of Georgia, Athens, GA 30602, USA

**Keywords:** *Brassica oleracea*, advanced backcross population, near isogenic introgression line, morphological traits, cauliflower, Plant Genetics and Genomics

## Abstract

To improve resolution to small genomic regions and sensitivity to small-effect loci in the identification of genetic factors conferring the enlarged inflorescence and other traits of cauliflower while also expediting further genetic dissection, 104 near-isogenic introgression lines (NIILs) covering 78.56% of the cauliflower genome, were selected from an advanced backcross population using cauliflower [*Brassica oleracea* var. *botrytis* L., mutant for *Orange* gene (ORG)] as the donor parent and a rapid cycling line (TO1434) as recurrent parent. Subsets of the advanced backcross population and NIILs were planted in the field for 8 seasons, finding 141 marker-trait associations for 15 leaf-, stem-, and flower-traits. Exemplifying the usefulness of these lines, we delineated the previously known flower color gene to a 4.5 MB interval on C3; a gene for small plant size to a 3.4 MB region on C8; and a gene for large plant size and flowering time to a 6.1 MB region on C9. This approach unmasked closely linked QTL alleles with opposing effects (on chr. 8) and revealed both alleles with expected phenotypic effects and effects opposite the parental phenotypes. Selected *B. oleracea* NIILs with short generation time add new value to widely used research and teaching materials.

## Introduction

*Brassica oleracea* is a species with remarkable morphological variation. Within the species, each of several “morphotypes” has an enlarged edible organ, including the lateral buds of Brussels sprouts (var. *gemmifera*), inflorescences of broccoli (var. *italica*), and cauliflower (var. *botrytis*), apical meristem and leaves of cabbage (var. *capitata*), bulbous epicotyl of kohlrabi (var. *gongylodes*), and leaves of kale and collard (var. *acephala*). In addition to these vegetables, rapid cycling lines which have been selected for short-generation time, self-compatibility, and absence of vernalization and seed dormancy have been considered a genetic standard within the species (Williams and Hill 1986). The diverse morphotypes and high nutritional content (Cao *et al*. 1996; Fahey *et al*. 1997; Sotelo *et al*. 2014), e.g. fiber, vitamins, and glucosinolates, have made *B. oleracea* a species with great economic importance.

Morphological variation within *B. oleracea* has been suggested to be a result of crops cross-breeding with wild types in the vicinity and/or being domesticated and selected in different environments (Dixon 2007). Nuclear DNA variation has suggested that the *B. oleracea* morphotypes could be divided into 3 groups. One group is composed of Chinese kale; another is composed of broccoli and cauliflower and the third includes the remaining morphotypes (Song *et al*. 1988, 1990; Cheng *et al*. 2016; Golicz *et al*. 2016). Regarding chloroplast genetic diversity, which is considered to be maternally inherited, broccoli and cauliflower have the same haplotype while cabbage, kohlrabi, and Chinese kale have another (Zhang *et al*. 2012).

To reveal the genetic control of morphological variation within *B. oleracea*, numerous genetic analyses have been done. Morphological variation has been dissected as leaf-, stem-, and inflorescence-related traits and mapped in various types of populations (Kennard *et al*. 1994; Lan and Paterson 2000, 2001; Sebastian *et al*. 2002). The enlarged inflorescences of cauliflower and broccoli are specifically of interest. In crosses between broccolis, QTLs were mapped for head- and leaf-traits (Brown *et al*. 2007; Walley *et al*. 2012; Lin *et al*. 2013). Genome-wide association (GWA) mapping in 174 cauliflower accessions suggested 24 significant associations for curd-related traits. In the same population, genomic prediction abilities ranged from 0.1 to 0.66 for various traits (Thorwarth *et al*. 2018). QTL mapping and GWA studies also revealed the genetic architecture of broccoli head and cauliflower curd under heat and various temperatures (Matschegewski *et al*. 2015; Hasan *et al*. 2016; Branham *et al*. 2017; Lin *et al*. 2018). With forward and reverse genetic analyses, candidate genes for curding phenotypes had been suggested to be *CAULIFLOWER* (*CAL*) and *APETALA1* (*AP1*), but these are not the only contributors to the phenotype (Kennard *et al*. 1994; Kempin *et al*. 1995; Lan and Paterson 2000; Purugganan *et al*. 2000; Smith and King 2000; Labate *et al*. 2006; Gao *et al*. 2007; Duclos and Björkman 2008; Sheng *et al*. 2019). QTL mapping of flowering time has been widely practiced in the species with candidate genes inferred from *Arabidopsis thaliana* (Bohuon *et al*. 1998; Rae *et al*. 1999; Lan and Paterson 2000; Axelsson *et al*. 2001; Lin *et al*. 2005, 2018; Okazaki *et al*. 2007; Razi *et al*. 2008; Li *et al*. 2015; Irwin *et al*. 2016; Branham *et al*. 2017). Additionally, methylation polymorphism has been reported to correlate with leaf morphology (Salmon *et al*. 2008).

Near isogenic lines (NILs), with single or small numbers of introgressed segments from a donor parent in a homogeneous genetic background, can serve as a good resource in both genetic mapping and breeding (Kooke *et al*. 2012). Since there are few (ideally one) introgressed segment(s) in each NIL, phenotypes due to QTLs on the segment(s) are rendered much more discrete than in F_2_ or backcross populations, often behaving as simple Mendelian factors (Paran and Zamir 2003). QTL mapping based on NILs can thus increase accuracy of QTL position and detect small-effect QTLs that might otherwise be obscured by larger-effect genes in more complex populations. In addition, because of the fixed genotype of NILs, they can be replicated in different environments to test interaction between genetic and environmental factors (Monforte and Tanksley 2000). By crossing NILs to the recurrent parent, fine mapping of specific QTLs toward their cloning is facilitated. A population of recombinant backcross substitution lines in *B. oleracea* (Ramsay *et al*. 1996) was employed for QTL mapping of flowering time and results suggested that more QTL could be found than in a doubled haploid population (Rae *et al*. 1999).

To understand the genetic architecture of morphological variation between cauliflower and rapid cycling line, we constructed an advanced backcross population. Cauliflower [*B. oleracea* var. *botrytis* L., mutant for *Orange* gene (ORG) (Lu *et al*. 2006)] was used as donor parent and a rapid cycling line (TO1434) (Williams and Hill 1986) as recurrent parent. Here, we selected lines each with 1 introgressed chromosomal segment from cauliflower in rapid cycling genetic background from the self-progenies of BC_4_F_1_ individuals. The selected near-isogenic introgression lines (NIILs) cover 78.56% of the cauliflower genome, enabling detailed phenotyping to map genes responsible for 15 morphological traits and evaluate the merit of these lines for dissecting complex phenotypes.

## Materials and methods

### Plant materials and population development

The mapping population was constructed using an inbred rapid cycling line (TO1434) as recurrent parent and cauliflower [*B. oleracea* var. *botrytis* L., mutant for *Orange* gene (ORG) (Lu *et al*. 2006)] as donor parent. TO1434 was pollinated by ORG to produce F_1_ hybrids, so that the progenies would have cytoplasm from TO1434. Then, TO1434 was used as pollen source for backcrosses to reach BC_4_F_1_. During population construction, plants were grown in a growth chamber with a 15.5-hour photoperiod and 23°C/20°C day/night temperature. Eight weeks after sowing, plants that had not formed flower buds were vernalized for 70 days with an 8-hour photoperiod and constant 4°C temperature before being returned to the growth chamber to flower. While few BC4 lines required vernalization (perhaps due to some inadvertent selection as they tended to be poor seed setters), these lines were included in the field trials and flowered very late.

BC_4_F_1_ lines with few introgressions were self-pollinated in greenhouse or growth chamber and their progenies genotyped by microsatellites (SSRs) in a targeted manner for NIL selection.

### DNA extraction

DNA of each individual was extracted from fresh leaves as reported in Paterson *et al*. (1993), with extraction buffer replaced by 0.35 M sorbitol, 0.1 M Tris, 0.005 M EDTA, 0.04 M sodium bisulfite and lysis buffer replaced by 0.2 M Tris at pH 8.0, 0.05 M EDTA, 2 M NaCl, 0.05 M CTAB.

### Genotyping-by-sequencing

Genotyping used genotyping-by-sequencing (GBS) for whole-genome scans, and SSRs (below) for targeted genotyping of specific segregating loci. GBS library construction followed a slightly modified multiplexed shotgun genotyping (MSG) (Andolfatto *et al*. 2011) procedure. Constructed libraries were sequenced using an Illumina Miseq. Sequencing data were analyzed using TASSEL 5 GBS v2 (Glaubitz *et al*. 2014). In TASSEL-GBS, the first 64 bps of each read are mapped on a reference genome (Parkin *et al*. 2014) to decide the position of the reads. SNP is called based on the alignment of reads. Heterozygosity at a locus is “called” (inferred) if the donor parent allele is present. Otherwise, a locus will be called as homozygous for the recurrent parent genotype.

### SSR markers and PCR genotyping

A total of 546 SSR markers were retrieved from published literature (Lowe *et al*. 2004; Piquemal *et al*. 2005; Burgess *et al*. 2006; Iniguez-Luy *et al*. 2008; Wang *et al*. 2012; Izzah *et al*. 2014; Shi *et al*. 2014). Since some marker names from Shi *et al*. (2014) conflict with those of Wang *et al*. (2012), markers chosen from the former are indicated with .j at the end. Each marker was BLASTed to the genome to reveal its physical location (Parkin *et al*. 2014). Polymerase chain reaction (PCR) was performed using 30 ng of DNA as template, 1 U Taq polymerase, 1 µL of 10 × PCR buffer (100 mM Tris-HCl at pH 9, 500 mM KCl, and 15 mM MgCl_2_), 1 µL of 2 mM dNTP, 1 µL of 25 mM MgCl_2_, 0.5 µL of 20 µM of each primer, with final reaction volume of 10 µL. PCR reactions were denatured at 95°C for 3 minutes, followed by 11 cycles of 95°C for 30 seconds, 55–65°C for 1 minute with 1°C increases at each cycle, and 72°C for 1 minute; then another 33 cycles of 95°C for 30 seconds, 55°C for 1 minute, and 72°C for 1 minute. The final cycle at 72°C was for 5 minutes, then samples were held at 4°C. Amplified fragments were analyzed in 10% polyacrylamide gels with silver staining.

### Phenotype evaluation

Self-pollinated progenies of advanced backcross populations, with multiple introgressions per line; and NIILs, with 1 introgression per line, were planted at the UGA Horticulture Farm, Watkinsville, GA, USA. Selfed progenies of the BC_4_ populations were planted in the field in 2014 spring, 2014 fall, 2015 spring, 2015 fall, and 2016 spring; and NILs were planted in 2017 spring, 2017 fall, and 2018 spring. Seedlings were started in a greenhouse and transplanted to the field when they were 3–4 weeks old. The families were planted in completely randomized designs with 5 replicates per lines. Block designs were implemented in 2017 fall and 2018 spring. In each season, recurrent parent TO1434 was included as control. While the donor parent was also grown, being much larger and later maturing it was much less meaningful for comparing to the lines that were converging on the genome of TO1434. Fifteen traits were evaluated which are listed below. Since we did not observe curd-like phenotypes in prior studies, the inflorescence architecture is approximated by bud and cluster number and cluster width. Phenotypes are recorded on the first day of flower opening, except budding date.

1. Leaf length: length of the largest leaf.
2. Lamina length: length of lamina of the largest leaf.
3. Lamina width: width of lamina of the largest leaf.
4. Blade shape: ratio between lamina width and lamina length.
5. Petiole length: length of petiole of the largest leaf.
6. Node number: number of nodes along main stem.
7. Plant height: length from ground to apex of plant.
8. Internode distance: plant height divided by node number.
9. Stem width: maximum width of stem.
10. Bud number: number of buds on the first cluster.
11. Cluster width: maximum width of the first cluster.
12. Cluster number: number of clusters.
13. Flower color: color of petals.
14. Budding time: days from transplanting to budding.
15. Flowering time: days from transplanting to flowering.

### Statistical analysis

Recombination frequencies in the BC_4_F_1_ generation were estimated by R/qtl (Broman *et al*. 2003). Introgression was inferred in cases where 2 consecutive SNP alleles were from the donor parent. Graphical genotypes are drawn by GGT 2.0 (van Berloo 2008). Evaluation of introgression frequencies relative to Mendelian expectations was tested by chi-squared tests in statistical software R (R Core Team 2016).

The differences between introgression families and the recurrent parent were revealed by Dunnett's 2-tailed t-test in SAS software (SAS Institute Inc. 2018). Single marker analyses were done in R/qtl (Broman *et al*. 2003). The significance threshold was set to LOD of 3, to mitigate the multiple-comparison problem. Filtration of significant markers adopted the method proposed by Szalma *et al*. (2007). If several markers on the same introgressed segment show significant association with phenotype, the most significant one was reported. For the co-segregation of multiple introgressions, the QTL location is examined as follows. First, if multiple families show significance for the trait and carry overlapping introgression, the introgression is considered to carry QTL. Second, if the co-segregation of introgressions is in single families, the most significant introgression is considered to carry QTL. Distribution of markers across the genome is visualized through PhenoGram (Wolfe *et al*. 2013).

Phenotypic variance explained by each locus was reported by taking the most significant marker as independent variable and phenotypic value as dependent variable in R (R Core Team 2016). Additive effects were estimated by half of the difference of phenotypic values between the homozygous donor and homozygous recurrent parent alleles. Dominance effects were estimated by the difference of phenotypic values between heterozygotes and the average of the 2 homozygote genotypes. To reach a finer resolution of markers detected on overlapping introgressions, individuals with the same introgressions were pooled together and compared to the recurrent parent. Phenotypic values of the introgressed families were reported as the mean of the phenotypes within each family.

## Results

**Fig. 1.**
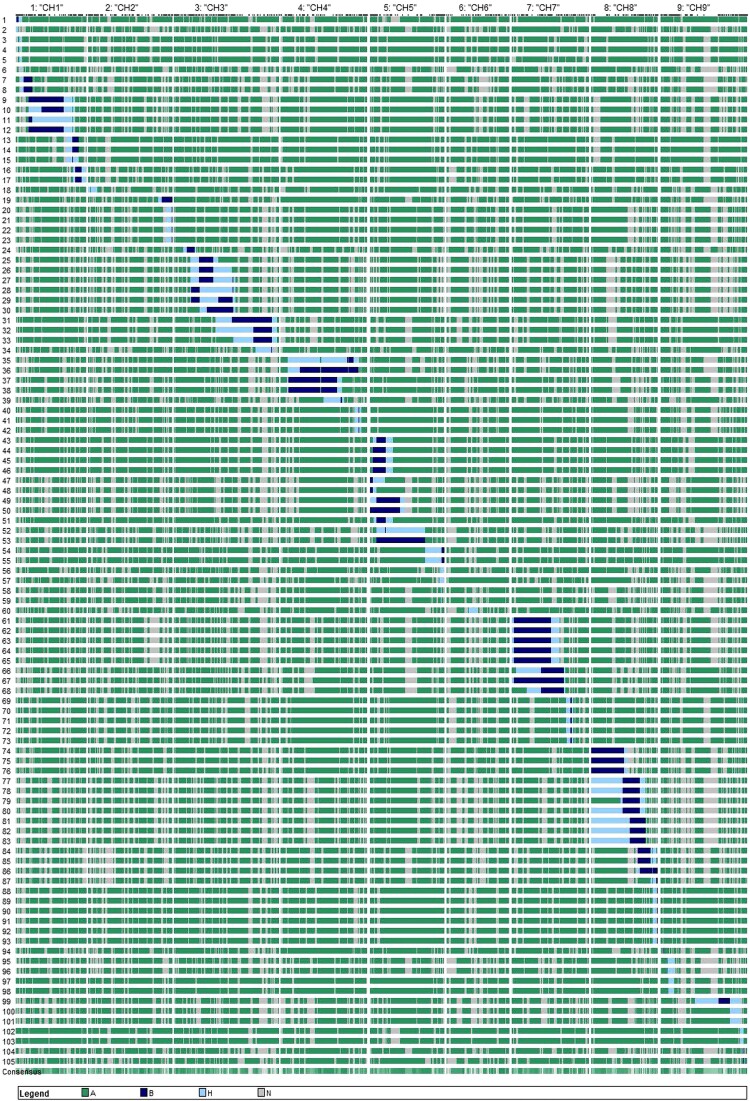
Graphical genotypes of cauliflower NIILs, with marker genotypes indicated in green (homozygous TO1434), light blue (heterozygous cauliflower), dark blue (homozygous cauliflower), or gray (missing data).

**Fig. 2.**
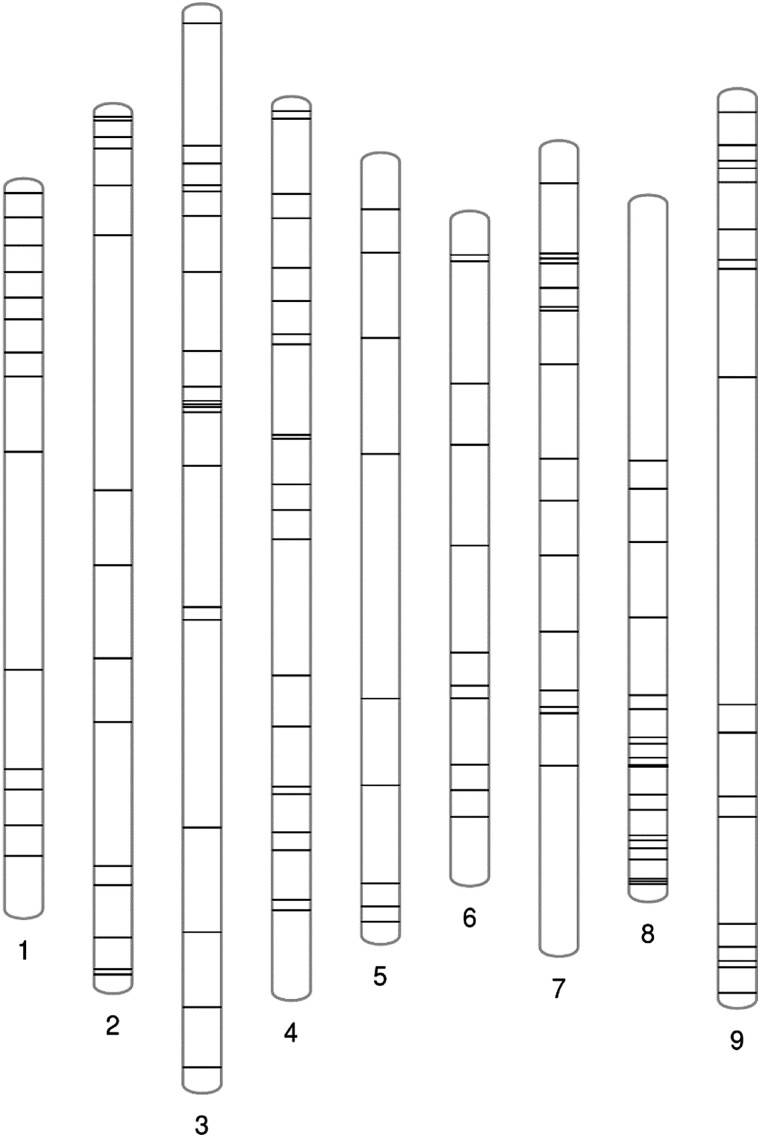
Distribution of SSR markers on 9 chromosomes.

From an advanced backcross population based on cauliflower genotype “Orange” (ORG), as donor parent and rapid cycling line (TO1434) as recurrent parent, a total of 91 BC_4_F_1_ individuals were genotyped by 908 informative SNPs (Supplementary Table 1). NIILs were selected from the selfed progenies of BC_4_F_1_ individuals in a targeted manner, identifying 104 lines with 1 fixed introgression per line that collectively cover 78.56% of the cauliflower genome (Fig. 1, Supplementary Table 2) by using SSR markers well distributed across the genome (Fig. 2).

### Phenotypic evaluation and QTL analyses

**Fig. 3.**
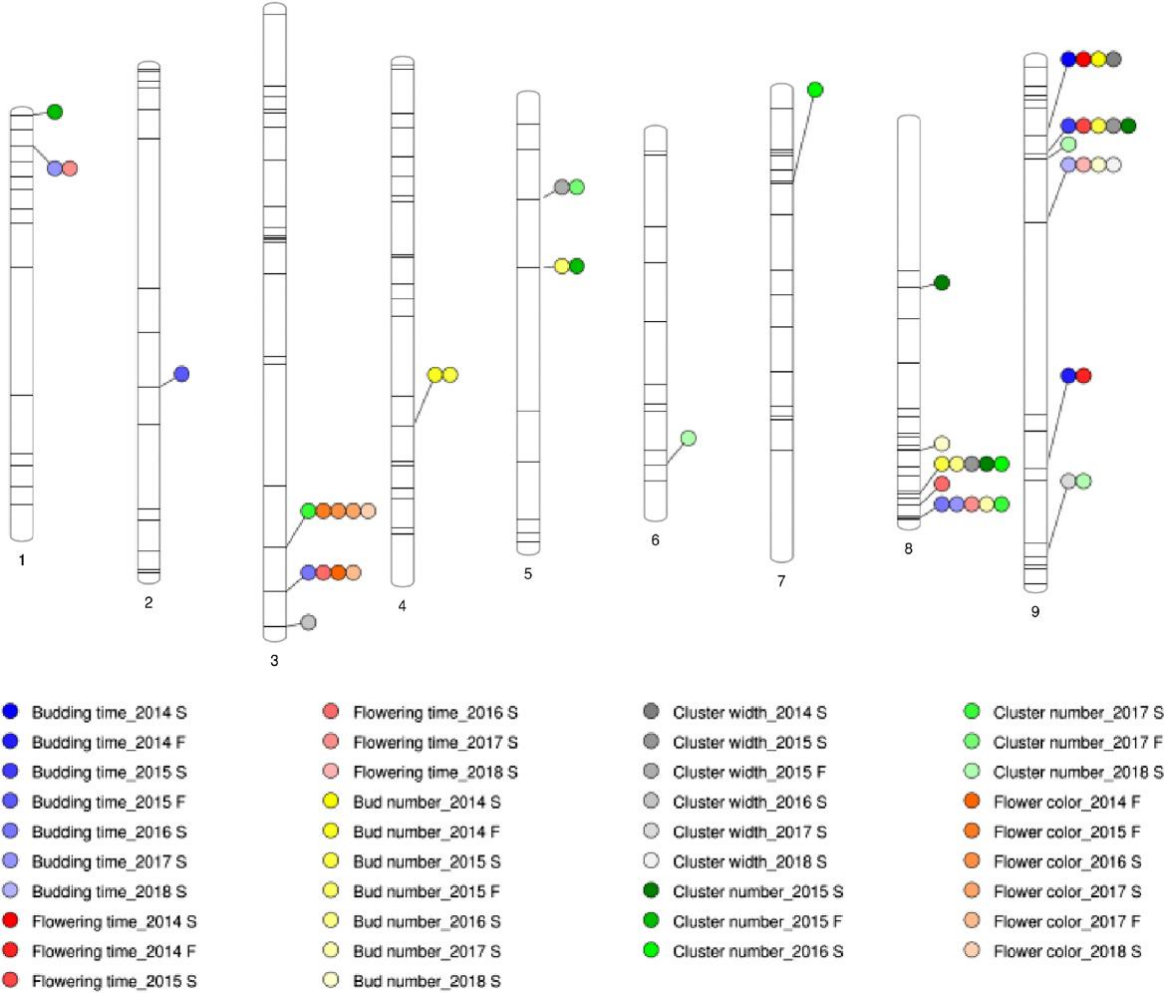
Distribution of SSR markers significantly associated with inflorescence traits.

**Fig. 4.**
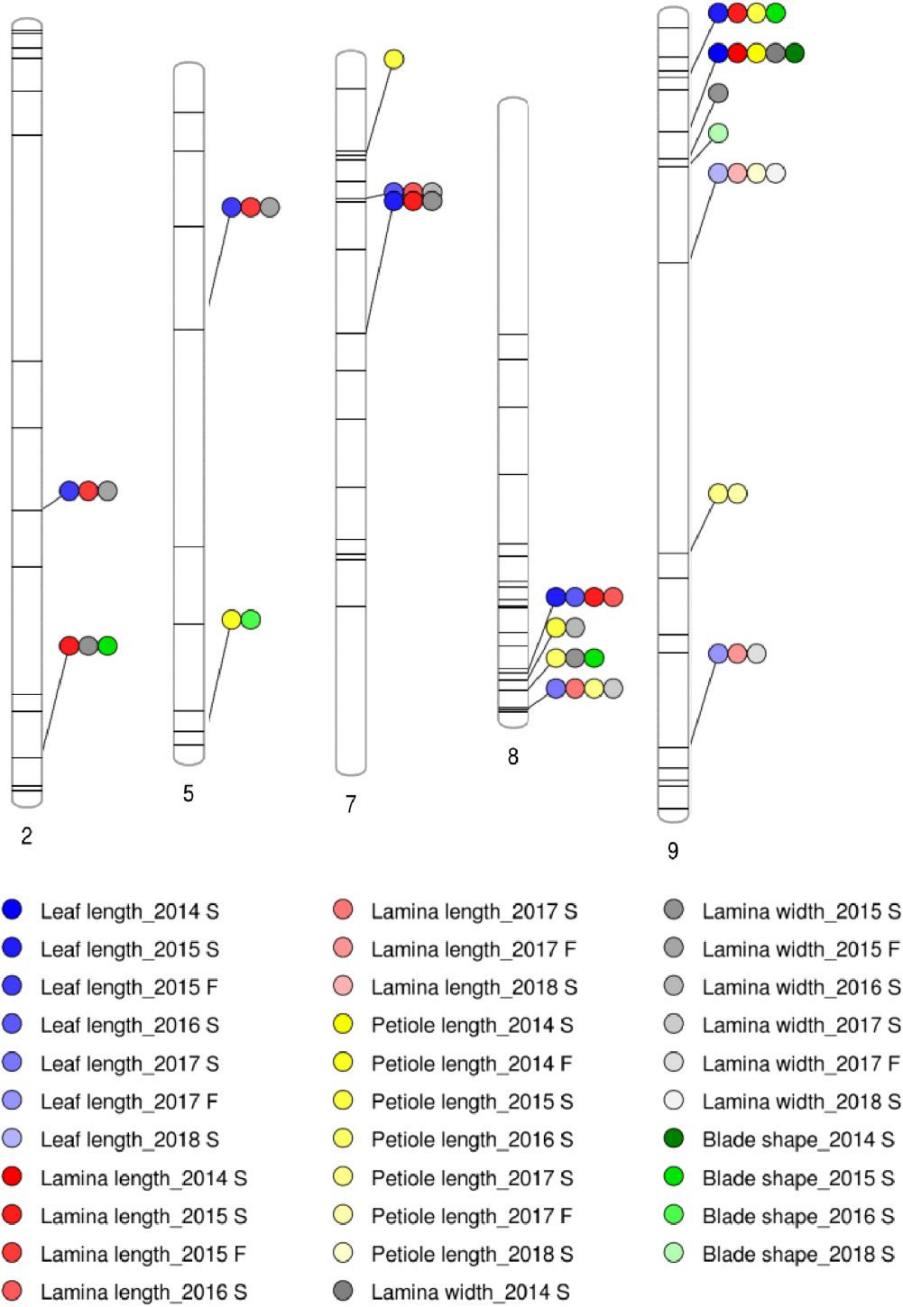
Distribution of SSR markers significantly associated with leaf traits.

**Fig. 5.**
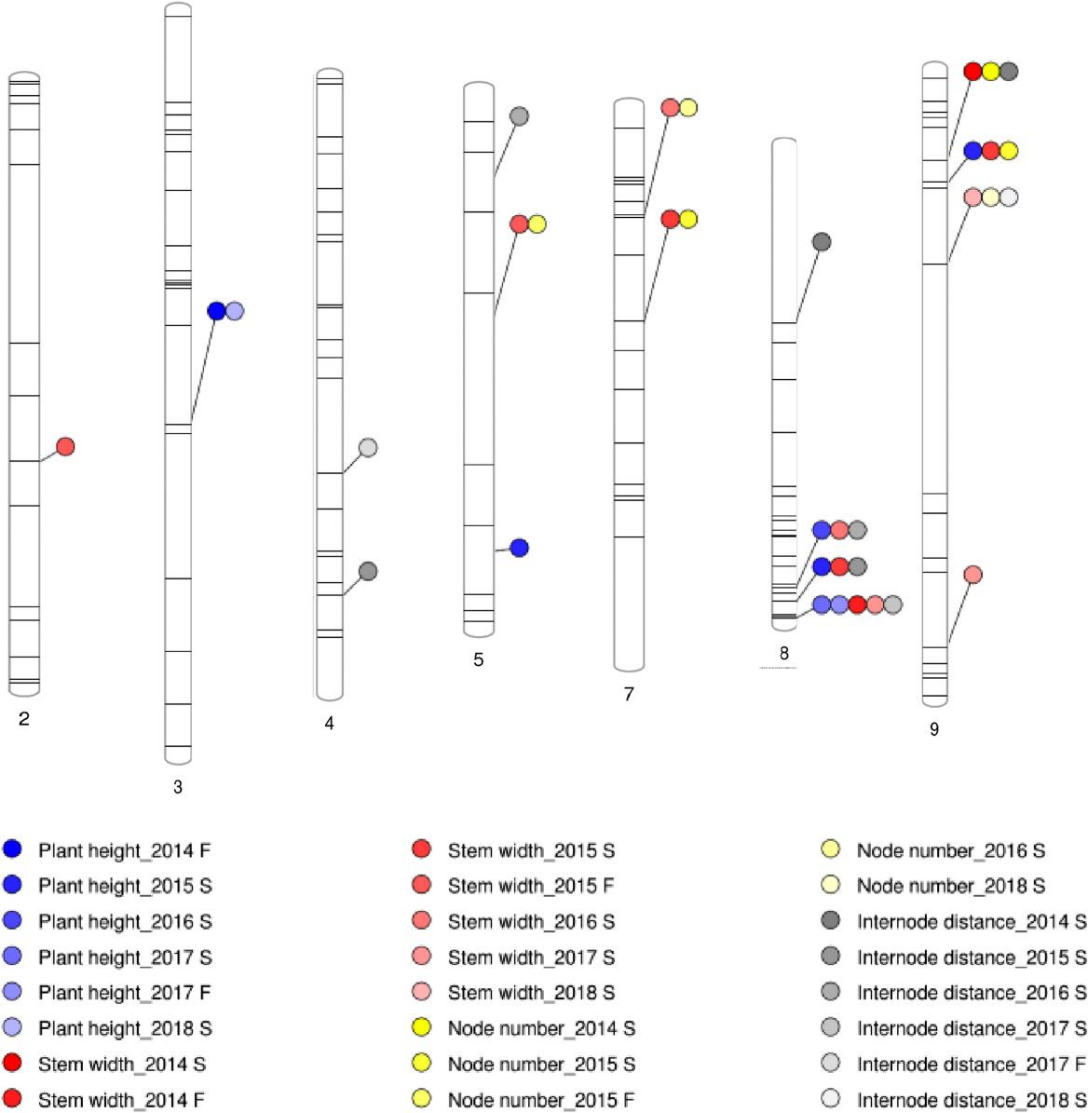
Distribution of SSR markers significantly associated with stem traits.

To dissect morphological variation between cauliflower and rapid cycling lines, we planted 2 types of populations in the field, including selfed progeny of advanced backcross lines with multiple introgressions per line from 2014 spring to 2016 spring (Table 1, Supplementary Tables 3–11); and NIILs with exactly 1 introgression per line from 2017 spring to 2018 spring (Table 2, Supplementary Tables 3–11). Fifteen traits were recorded on the day that the first flower opened. Cluster number was not recorded in 2014 spring. Across 8 seasons, we found 141 marker-trait associations (Figs. 3–5). Flower color segregated in the field as a qualitative trait.

**Table 1.** Summary of populations planted for field evaluation (advanced backcrosses).

	Season	Generation	BC_4_F_1_-derived families	No. of individual lines tested	Number of SSRs genotyped	Genome coverage of introgressed segments
2014	Spring	BC_4_F_2_	29	145	97	783/908 (86%)
	Fall	BC_4_F_3_	15	103	65	550/1000 (55%)
2015	Spring	BC_4_F_3_	25	232	87	736/1012 (73%)
	Fall	BC_4_F_3_	29	235	89	768/1012 (76%)
2016	Spring	BC_4_F_3_	22	295	70	600/1013 (59%)

**Table 2.** Summary of populations planted for field evaluation (near isogenic introgression lines (NIIL)).

Season	NIIL	1 unfixed introgression	2 introgressions	Genome coverage (%)
2017 Spring	26	0	1	61
2017 Fall	24	0	3	65
2018 Spring	36	1	3	76

### Flower color

**Fig. 6.**
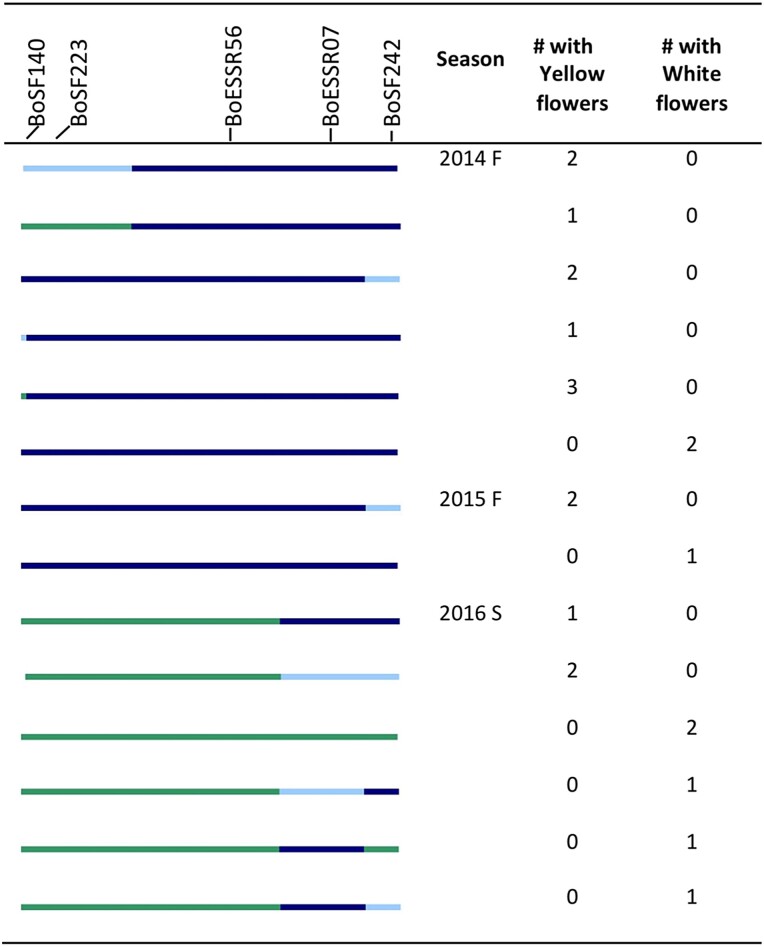
Segregation of flower color in different seasons and BC4 families. Line color at left indicates DNA marker genotypes in salient introgression region, distinguishing homozygosity for To1434 allele (green) from heterozygous (light blue) or homozygous (dark blue) introgression from cauliflower. Gray indicates missing data, and recombinants are inferred to be midway between consecutive markers.

While TO1434 has white petals, cauliflower has yellow petals. Segregation of flower color was observed in 2014 fall, 2015 fall and 2016 spring (Figs. 3 and 6). Comparisons between phenotypes and genotypes suggested that the genomic region determining flower color was between nucleotides 55,802,077 (BoESSR073) and 60,314,017 (BoSF2423) on C3. The region includes *CAROTENOID CLEAVAGE DIOXYGENASE 4* (*BoCCD4*), located at 56,605,961–56,607,751, which was recently identified as the gene controlling flower color in *B. oleracea* and its amphidiploid relatives *B. napus* and *B. carinata* (Zhang *et al*. 2015; Han *et al*. 2019).

### Cauliflower allele(s) conferring reduced plant size

Markers CB10092, BoSF2819, BoSF2612, BoE836, and BrBAC260, detecting the same introgressions at the end of C8, were associated with numerous plant size traits in 2014 fall, 2015 spring, 2016 spring, 2017 spring, and 2017 fall. This region has shown association with all traits except node number and is linked to negative additive effects on leaf-, stem-, and inflorescence-traits and positive additive effects on budding and flowering time. Thus, in contrast to the larger size of the parental line, the cauliflower allele in this region is contributing to reduced plant size and delayed flowering time.

**Fig. 7.**
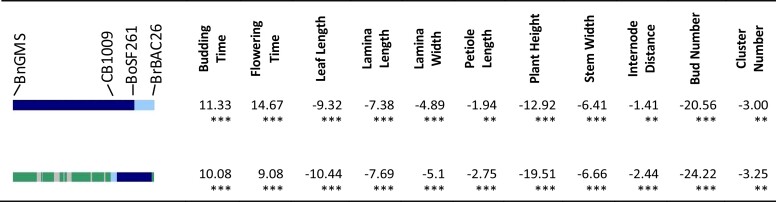
Introgressions at the end of chromosome 8 carried by lines O#34.5.13 and O#61.4.15 in 2017 spring. Effect of cauliflower introgression was reported as the difference between the mean of introgressed individuals and To1434. Significance was reported by *, with *P*-value <0.05 as *, <0.01 as **, <0.001 as ***. Line color at left indicates DNA marker genotypes in salient introgression region, distinguishing homozygosity for To1434 allele (green) from heterozygous (light blue) or homozygous (dark blue) introgression from cauliflower. Gray indicates missing data, and recombinants are inferred to be midway between consecutive markers.

These marker-trait associations at the end of C8 were confirmed by 2 NIILs derived from O#34 and O#61 (Fig. 7). The introgressions carried by the 2 NIILs overlapped from 37,649,250 to the chromosome end. These 2 families had small plant size in the field in 2017 spring and fall and 2018 spring. After transplanting, the plants started to show stressed and dwarf phenotypes, with small, yellowish and thickened leaves, thin stems, and long vegetative growth. In 2017 fall, similar phenotypes were observed at the beginning of the season. Interestingly, the stressed and dwarf phenotypes disappeared by flowering. In 2018 spring, the phenotypes were so severe that only 1 plant survived to flower.

**Fig. 8.**
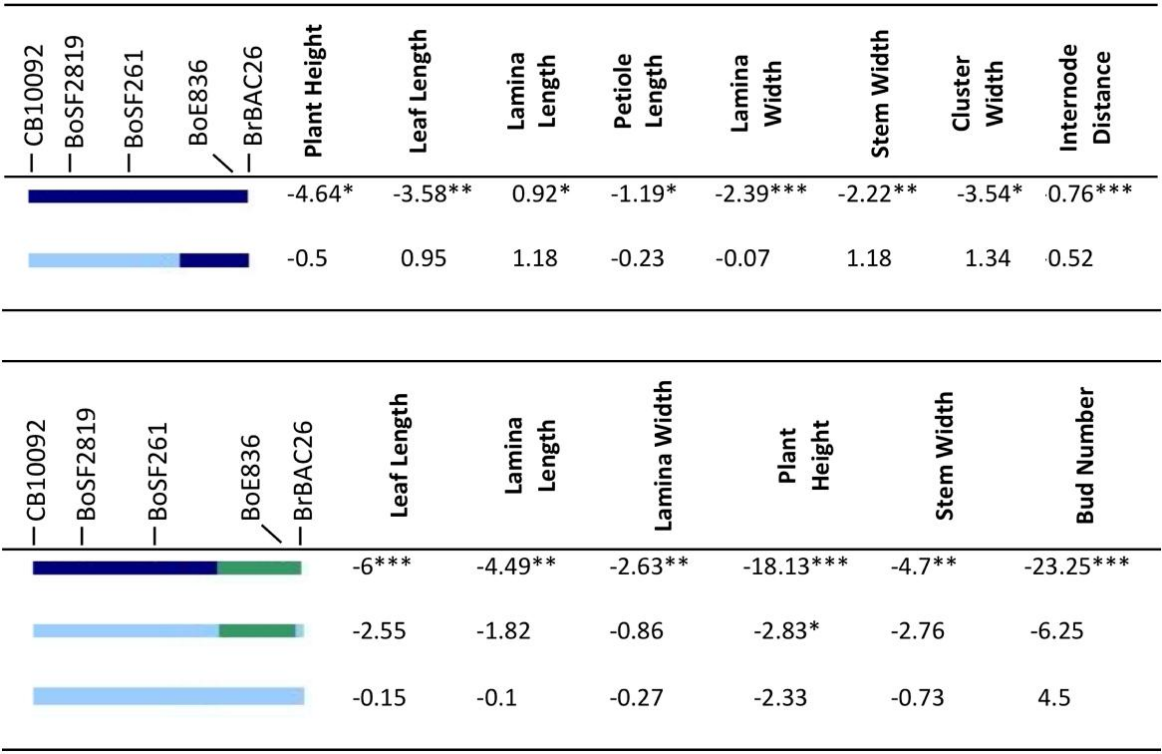
a) (Upper) Segregation of the chromosome 8 terminal region in 2015 spring. Effect of cauliflower introgression was reported as the difference between the mean of introgressed individuals and To1434. Significance was reported by *, with *P*-value <0.05 as *, <0.01 as **, <0.001 as ***. Line color at left indicates DNA marker genotypes in salient introgression region, distinguishing homozygosity for To1434 allele (green) from heterozygous (light blue) or homozygous (dark blue) introgression from cauliflower. Gray indicates missing data, and recombinants are inferred to be midway between consecutive markers. b) (Lower) Segregation of the chromosome 8 terminal region in 2016 spring. Effect of cauliflower introgression was reported as the difference between the mean of introgressed individuals and To1434. Significance was reported by *, with *P*-value <0.05 as *, <0.01 as **, <0.001 as ***. Line color at left indicates DNA marker genotypes in salient introgression region, distinguishing homozygosity for To1434 allele (green) from heterozygous (light blue) or homozygous (dark blue) introgression from cauliflower. Gray indicates missing data, and recombinants are inferred to be midway between consecutive markers.

Selfed progenies planted in 2015 and 2016 spring showed segregation for plant size traits. Based on single marker analysis, CB10092 (38,579,021), BoSF2819 (39,053,207), and BoSF2612 (39,792,591) showed significant association with traits related to leaf and plant size. In 2015 spring, lines carrying homozygous introgression at 38,579,021–41,244,859 showed significant differences from TO1434 in stem- and leaf-traits (Fig. 8a). Lines carrying homozygous introgression at BoE836 (41,048,747) and BrBAC260 (41,244,859) did not have any significant differences from TO1434. In 2016 spring, lines carrying homozygous introgression at CB10092, BoSF2819, and BoSF2612 were significantly different from TO1434 in leaf size, plant size, and bud number (Fig. 8b).

Comparison of these segregating individuals suggests that the candidate region for these plant size traits is from 37,649,250–41,048,747. In 2015 spring, most families carrying homozygous introgression in this region showed stressed phenotypes, except O#34.4. In 2016 spring, individual O#159.3.4 with homozygous introgression at CB10092 and BoSF2819 and homozygous recurrent parent genotype at BoSF2612 showed stress phenotypes in the field. The phenotypes and genotype of this individual indicate that the putative region of the causal gene is C8: 37,649,250–39,792,591, a region in which 344 of 391 genes are polymorphic between parents. Forty-one genes in this region have *A. thaliana* orthologs annotated as response to stress. One particularly intriguing candidate gene, Bo8g108050 (C8:38,486,219–38,486,824), is orthologous to *A. thaliana* AT1G12610, with phenotypes related to late flowering, dwarfism, and salt tolerance (Magome *et al*. 2004, 2008; Kang *et al*. 2011). Indels and SNPs in the coding and promoter regions differentiate Bo8g108050 between the 2 parents.

### QTL conferring large plant size and late flowering time

In the cauliflower population, DNA markers Ol10.D08, BoSF2364.j, BoSF2389.j, BoSF0383.j, and BoESSR484 detected the same introgressions and have been associated with leaf-, stem-, inflorescence-traits, and flowering time in 2014, 2015, and 2018 spring. In the 3 seasons, most of these markers were linked to positive additive effects suggesting that the cauliflower allele contributes to large plant size and late flowering. BoESF2364.j associated with internode distance in 2014 spring and BoESSR484 associated with bud number in 2018 spring are exceptions, with marker-trait associations indicating negative additive effects.

**Fig. 9.**
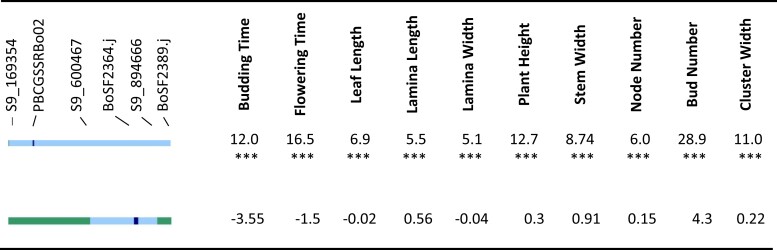
Effects of chromosome 9 introgression into families 10.4 and 147.2 in 2015 spring. Effect of cauliflower introgression was reported as the difference between the mean of introgressed individuals and To1434. Significance was reported by *, with *P*-value <0.05 as *, <0.01 as **, <0.001 as ***. Line color at left indicates DNA marker genotypes in salient introgression region, distinguishing homozygosity for To1434 allele (green) from heterozygous (light blue) or homozygous (dark blue) introgression from cauliflower. Gray indicates missing data, and recombinants are inferred to be midway between consecutive markers.

In 2015 spring, a family was evaluated, O#10.4, carrying 2 introgressions, with one on C1 and another on C9. The introgression on C9 ranged from 1,548,569 to 10,357,793. This family has large plant size and late flowering time, with significant differences in multiple leaf-, stem-, inflorescence-traits, and flowering time traits from TO1434. Another family, O#147.2, with introgression in 6,004,676–8,946,661 and confirmed to be homozygous at BoSF2364.j (7,963,701), did not show significant difference from TO1434 in any trait in 2015 spring (Fig. 9).

**Fig. 10.**
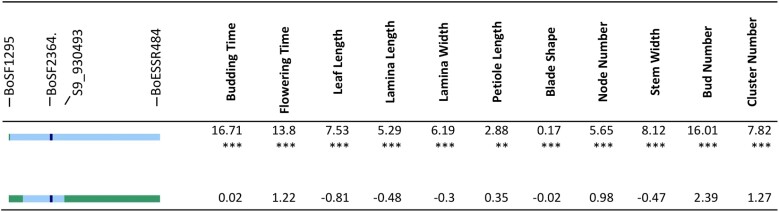
Effects of chromosome 9 introgression into families 134.18 and 147.10.2 in 2018 spring. Effect of cauliflower introgression was reported as the difference between the mean of introgressed individuals and To1434. Significance was reported by *, with *P*-value <0.05 as *, <0.01 as **, <0.001 as ***. Line color at left indicates DNA marker genotypes in salient introgression region, distinguishing homozygosity for To1434 allele (green) from heterozygous (light blue) or homozygous (dark blue) introgression from cauliflower. Gray indicates missing data, and recombinants are inferred to be midway between consecutive markers. Effect of cauliflower introgression was reported as the difference between the mean of introgressed individuals and To1434. Significance was reported by *, with *P*-value <0.05 as *, <0.01 as **, <0.001 as ***. Line color at left indicates DNA marker genotypes in salient introgression region, distinguishing homozygosity for To1434 allele (green) from heterozygous (light blue) or homozygous (dark blue) introgression from cauliflower. Gray indicates missing data, and recombinants are inferred to be midway between consecutive markers.

A family, O#134.18, carrying introgression at the region was planted in 2018 spring. With 1 heterozygous introgression from 5,103,975 to 16,943,553, the lines had later flowering time, larger leaf size, and stem width than TO1434. A sibling line of O#147.10.2 with the same introgression and also homozygous at BoSF2364.j, O#147.10.2, was planted 2018 spring and did not show significantly different phenotypes from TO1434 (Fig. 10).

**Fig. 11.**
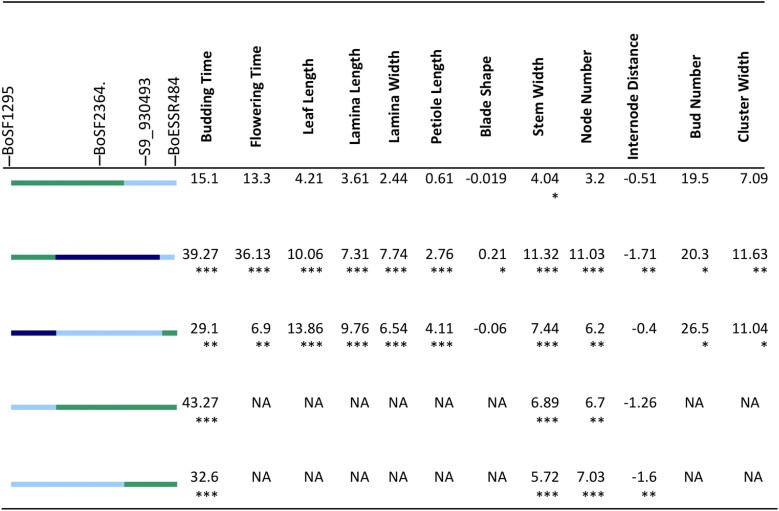
Segregation of a candidate region on chromosome 9 in 2014 spring. Effect of cauliflower introgression was reported as the difference between the mean of introgressed individuals and To1434. Significance was reported by *, with *P*-value <0.05 as *, <0.01 as **, <0.001 as ***. Line color at left indicates DNA marker genotypes in salient introgression region, distinguishing homozygosity for To1434 allele (green) from heterozygous (light blue) or homozygous (dark blue) introgression from cauliflower. Gray indicates missing data, and recombinants are inferred to be midway between consecutive markers.

Based on 2015 and 2018 spring, the putative region for large plant size and late flowering time might be the overlapping region between O#10.4 and O#134.18, from 4,242,853 to 10,357,793. This is defined by the closest SSR marker having homozygous recurrent parent genotypes in either one of the families. Since the progenies of O#147 did not show significant differences in both seasons, this might exclude 6,004,676–8,946,661 from the candidate region. In 2014 spring, multiple families carried introgression at the region. The individuals having the same genotype at the region were pooled together. Significant differences between introgression lines and TO1434 suggest that the putative region is in 4,242,853–9,838,674 but excluding 6,004,676–8,946,661 (Fig. 11).

There are 820 genes within C9: 4,242,853–10,357,793, among which 723 have sequence polymorphism between cauliflower and TO1434. Excluding the region of 6,004,676–8,946,661, 7 known flowering time or flower development genes are polymorphic between parents (Supplementary Table 12).

### QTL on C7 associated with increased leaf and stem size

On C7, 2 markers, BoSF2784 and BoSF1581, are linked to leaf length, lamina length, lamina width, stem width, and node number in 2015 and 2016 spring (Supplementary Table 13; Figs. 4 and 5). The two markers detect the same introgressions. Both markers are linked to positive additive effects, which suggests the cauliflower allele contributes to increased leaf and stem size. However, the NILs carrying the introgression at the region did not show significant differences in stem- and leaf-traits from TO1434 in 2017 and 2018 spring.

### Flowering time traits

Four markers on C1, C2, C3, and C9 are associated with budding time, explaining 5.9–69.45% of phenotypic variation. Three of the four markers, except the one on C2, are also associated with flowering time, explaining 5.3–47.56% of phenotypic variation (Supplementary Table 13; Fig. 3).

### Leaf traits

Markers on C2, C5, and C9 explain 10.47–16.04% of phenotypic variation of leaf length, 6.1–15.96% of phenotypic variation of lamina length, and 6.44–12.94% of phenotypic variation of lamina width. In addition to leaf size, 3 of these markers are also associated with stem width, explaining 11.81–21.43% of phenotypic variation. All markers are linked to positive additive effects, which suggests that the cauliflower allele contributes to increased leaf size and stem width. Three markers on C5, C7, and C9 are associated with petiole length, explaining 7.34–18.74% of phenotypic variation. BoSF052 shows significant association with petiole length in 2017 spring and fall. Two markers, BrSF177 on C2 and BoSF2390 on C5, are associated with blade shape in 2015 and 2016 spring, respectively, explaining 6.22 and 8.68% of phenotypic variation (Supplementary Table 13; Fig. 4).

### Stem traits

BoSF1408 on C3 and BrSF0623.j on C5 are associated with plant height. BoSF1408 shows significant association in 2014 fall and 2018 spring. CB10027 is associated with node number in 2015 fall, explaining 15.94% of phenotypic variation. Four markers, on C4, C5, and C8, are associated with internode distance, explaining 5.55–12.9% of phenotypic variation. One family, O#9.8.11, carrying introgression at the region, C4:4,643,964–48,113,158, showed significantly longer internode distance than TO1434 in 2017 fall, consistent with the significant association between the trait and BoSF2863 (Supplementary Table 13; Fig. 5).

### Inflorescence traits

Three markers on C4, C5, and C8 are associated with bud number, explaining 8.12–22.61% of phenotypic variation. Ol10D03 on C4 shows significant association in 2014 fall and 2015 spring and is linked to positive additive effects in both seasons (Supplementary Table 13; Fig. 3).

Three markers on C3, C5, and C9 are associated with cluster width, explaining 8.84–22.57% of phenotypic variation in each season. Eight markers on C1, C3, C5, C6, C7, C8, and C9 are associated with cluster number in various seasons, explaining 5.31–19.85% of phenotypic variation. On C5, CB10027 and cnu149 detect linked introgression in 2015 and 2017 fall with positive additive effect which suggests the cauliflower allele is contributing to more clusters (Supplementary Table 13; Fig. 3).

### Regions show significance in introgression lines

In the cauliflower population, 9 and 5 families showed significant differences from TO1434 in various traits in 2017 and 2018 spring, respectively; O#9.11.16 had shorter petiole length and O#9.8.11 had longer internode distance than TO1434 in 2017 fall (Supplementary Table 13). Between seasons, O#109.2.17, which carries introgression on chromosome 9, 50,119,921–51,943,054, had shorter internode distance than TO1434 in 2017 and 2018 spring. While most of these lines are consistent with the results of single marker analyses, some lines might suggest additional candidate regions for traits, including a region on C7 in O#19.15.12 for budding time; C4 in O#151.3.11 for stem width; C7 in O#8.2.6 for blade shape; C2 in O#9.11.16 for petiole length; and C9 in O#109.2.17 for internode distance.

## Discussion

In the cross between cauliflower genotype “Orange” and rapid cycling line TO1434, we have investigated 15 morphological traits and found 141 marker-trait associations in advanced backcross populations and/or NIILs. While different loci associated with the same trait under various environments might suggest interaction between genotype and environment, loci being repeatedly detected across seasons might indicate environmental stability. In addition to major QTLs on C7, C8, and C9, we found 4 chromosomal regions consistently associated with specific traits in various environments. A region on C3 indicated by BoSF1408 is associated with plant height in 2014 fall and 2018 spring; a region on C4 indicated by Ol10D03 is associated with bud number in 2014 fall and 2015 spring; a region on C5 indicated by CB10027 and cnu149 is associated with cluster number in 2015 fall and 2017 fall; and a region on C9 indicated by BoSF052 is associated with petiole length in 2017 spring and fall.

In published QTL studies, the major C9 QTL has been widely discovered, which might suggest that it is a common variant within the species (Alonso-Blanco *et al*. 2009). The region on C9 coincides with flowering QTL found in various crosses and mapping populations, including DH and backcross substitution lines from a cross between *B. oleracea* var. *alboglabra* (A12DH) and *B. oleracea* var. *italica* (GDDH33) (Bohuon *et al*. 1998; Rae *et al*. 1999); 3 F_2_ populations using a rapid cycling line as the common parent and *B. oleracea* var. Cantanese, *B. oleracea* var. Pusa Katki, and *B. oleracea* var. Bugh Kana as the other parents (Lan and Paterson 2000); and an F2 population from a cross between a doubled haploid line from A12DH × GDDH33 and a rapid cycling line (Axelsson *et al*. 2001). The region is also associated with inflorescence and plant size traits in 3 F2 populations (Lan and Paterson 2000, 2001) and DH populations from crosses between 2 broccoli DH lines GDDH33×MarDH34 (Walley *et al*. 2012).

Lines carrying cauliflower introgression on C8:37,649,250–41,048,747 have small plant size. One gene in this region, Bo8g108050 (C8:38,486,219–38,486,824), is orthologous to *A. thaliana* AT1G12610, also known as *DWARF AND DELAYED FLOWERING* 1 (*DDF1*) (Magome *et al*. 2004). Dominant Arabidopsis mutants with overexpression of this gene are late flowering, dwarfed and more salt-, freezing-, drought-, and heat-tolerant than the wild type (Magome *et al*. 2004, 2008; Kang *et al*. 2011). Under long days, the mutant flowers later than under short days (Magome *et al*. 2004). Loss of function of the gene results in longer primary roots under high salinity stress than wild type (Magome *et al*. 2008). Overexpression of *DDF1* in blueberry can enhance freezing-tolerance without affecting plant growth or flowering (Song and Gao 2017). Our Brassica introgression lines show delayed flowering and dwarfism that is similar to overexpression of *DDF1* in *A. thaliana*. However, our lines show stressed phenotypes after transplantation to the field—if *DDF1* is the candidate gene, this might suggest that instead of being abiotic-stress tolerant such as *ddf1* mutants of *A. thaliana*, the introgression lines (and therefore the cauliflower allele) are more sensitive to abiotic stress than the wild-type (T01434 allele).

In addition to a 3.4 MB candidate region of C8 associated with small plant size (37,649,250–41,048,747), we found an additional region responsible for reduced bud number. The marker-trait associations with bud number may have been neglected previously because of the large-effect QTL at the end of C8. In 2017 spring and 2018 spring, clusters of markers on C8: 29,795,106–34,147,843 showed significant association with negative additive effects on bud number. The region is covered by NIL family O#34.5.13 and O#121.4.14 in 2017 spring and only by O#121.4.14 in 2018 spring. In 2018 spring, the region shows significant association with bud number within O#121.4.14, which suggests that 2 regions on C8 might contribute to decreased bud number.

Co-localization of QTL might be the result of either pleotropic effects of single genes or close linkage of multiple genes (Mauricio 2001). For example, a major QTL on C9 associated with late flowering time and larger plant size might be the result of long vegetative growth, with delayed flowering permitting the plant to grow to larger size. We found 10 regions that are associated with multiple traits including BrBAC002 on C1; BoSF2279 and BrSF177 on C2; BoSF2423 on C3; CB10027 on C5; BoSF1581 and BoSF2784 on C7; from CB10092 to BoE836 on C8; from Ol10.D08 to BoESSR484; BoESSR901; BoSF0347.j and BoSF0654.j on C9.

In summary, a largely genome wide panel of NIILs together with advanced backcross lines from which they were produced, permit better resolution in dissecting QTLs than conventional F2, backcross or RIL populations, as exemplified by mapped loci in a 4.5 MB region on C3 for flower color; 3.4 MB region on C8 for small plant size; and 6.1 MB region on C9 for large plant size and flowering time. Selected NIILs with short generation time add new dimensions to facile and well established research and teaching materials for *B. oleracea*.

## Supplementary Material

jkad163_Supplementary_Data

## Data Availability

Genotypes of BC4F1 are in Supplementary Table 1. Genotypes of NIILs are in Supplementary Table 2, with individual lines identified based on 2–5 digit numbers reflecting the BC1-derived line, subsequent plant number(s) and generation that reached near isogenicity. Phenotypes of all seasons are in Supplementary Table 3. Genotypes of field samples are in Supplementary Tables 4–11. Single marker analyses are in Supplementary Table 12, and Dunnet's test of introgression families from 2017 spring to 2018 spring in Supplementary Table 13. Supplemental material available at G3 online.
